# Perioperative outcomes of robotic versus laparoscopic distal gastrectomy for gastric cancer: a meta-analysis of propensity score-matched studies and randomized controlled trials

**DOI:** 10.1186/s12893-022-01881-9

**Published:** 2022-12-14

**Authors:** Tao Sun, Yinghua Wang, Yan Liu, Zhanyu Wang

**Affiliations:** 1 Department of Internal Medicine, The Second Affiliated Hospital of Dalian Medical University, Dalian, China; 2 Department of Oncology, The Second Affiliated Hospital of Dalian Medical University, Dalian, China; 3 Department of General Surgery, The Second Affiliated Hospital of Dalian Medical University, No. 467, Zhongshan Road, Shahekou District, Dalian, 116023 Liaoning China

**Keywords:** Gastric cancer, Laparoscopic distal gastrectomy, Meta-analysis, Robotic distal gastrectomy

## Abstract

**Background:**

Da Vinci robotic surgery system, a novel type of surgery, was widespread in surgical field. However, the perioperative outcomes of robotic distal gastrectomy (RDG) are still controversy, despite several observational studies and randomized controlled trials (RCT) had been reported. Therefore, we performed a meta-analysis of propensity score-matched (PSM) and RCT studies to evaluated the perioperative feasibility and safety of RDG.

**Methods:**

Studies were systematically searched in PubMed, Web of Science, Cochrane Library, and Embase database, and screened according to the defined limitations. The quality of PSM studies and RCT studies were respectively assessed by ROBINS-I and Cochrane risk-of-bias tool. Extracted data were analyzed by Review Manager 5.4.

**Results:**

7 PSM studies and 1 RCT with a total of 2763 patients were included in this analysis. The longer operative time (MD = 31.42, 95% CI [22.88, 39.96], p < 0.00001), less blood loss (MD = − 25.89, 95% CI [− 36.18, − 15.6], *p* < 0.00001), more retrieved lymph nodes (MD = 3.46, 95% CI [2.94, 3.98], *p* < 0.00001), shorter time to first flatus (MD = − 0.08, 95% CI [− 0.13, − 0.02], *p* = 0.006) and liquid intake (MD = − 0.13, 95% CI [− 0.22, − 0.05], *p* = 0.002) were observed in RDG group compared with LDG group. There are no statistically significant in time to start soft diet, postoperative hospital stays, overall complications, complications Grade I–II, complications Grade ≥ III, anastomotic leakage, bleeding, intra-abdominal bleeding, intraluminal bleeding, ileus, abdominal infection, delayed gastric emptying and wound complications.

**Conclusions:**

RDG showed less blood loss and more retrieved lymph nodes, revealed less time to first flatus and liquid intake after operation. But the operative time was longer in RDG group than in LDG. The incidence rate of postoperative complications was comparable between RDG and LDG.

## Introduction

Gastric cancer (GC) has been identified as one of the most common cancers, the incidence of which is only second to lung cancer in China [1]. Radical gastrectomy with regional lymph node dissection is always the standard procedure for patients with GC [2]. At present, laparoscopic gastrectomy has been become the mainstream surgical method due to its advantages of less invasiveness, less pain, better cosmetic effect, faster recovery, and shorter hospital stays compared with open gastrectomy [3]. Although, 3D-laparoscopy can provide clear stereoscopic imaging effects, the limitations in straight instruments, amplified tremor, and the uncomfortable position of surgeons are still problems should not to be neglected [4]. The appearance of da Vinci robotic surgery system solved these problems well [5]. In recent years, this new type of surgery gradually gained the favor of surgeons, because of its 3D high-definition vision, multi-degree-of-freedom rotatable wrist device, tremor filtration, better ergonomics, and remote surgical consultation [6]. However, several high-quality research should be needed to prove the short-term perioperative outcomes and long-term surgical outcomes of robotic surgery.

From the robotic gastrectomy (RG) first be reported by Hashizume et al. in 2002 [7] up to now, several studies have compared the perioperative outcomes of RG with laparoscopic gastrectomy (LG) for patients diagnosed as GC [7–10]. Of which, many studies mixed distal gastrectomy, proximal gastrectomy, and total gastrectomy together for comparation [10, 11]. This can lead to very serious confounding bias, and affects the accuracy of almost all the perioperative outcomes, especially in the terms of operative time, blood loss, numbers of retrieved lymph nodes and the incidence of anastomotic leakage. Therefore, we performed a meta-analysis focused on distal gastrectomy for patients diagnosed as GC. Hitherto, only one meta-analysis of non-randomized controlled trials (non-RCT) compared robotic distal gastrectomy (RDG) with laparoscopic distal gastrectomy (LDG) for GC has been reported by Gong et al. in 2022 [12]. However, several studies being included are small sample volume studies and initial results in learning curve of robotic surgery [13–17], which are the major reason for high heterogeneity of many outcomes. In the hierarchy of research designs, the results of RCTs are considered the highest level of evidence. Propensity score-matched (PSM) analysis remove the confounding factors and overcome possible selection bias in observational studies, improve the quality of evidence approach to the level of RCT [18]. In order to make a high-quality comparison on the safety and feasibility of RDG versus LDG, we performed this meta-analysis only included PSM and RCT studies compared RDG with LDG for patients with GC.

## Methods

### Protocol and registration

This meta-analysis has been reported in line with PRISMA (Preferred Reporting Items for Systematic Reviews and Meta-Analyses) and AMSTAR (Assessing the methodological quality of systematic reviews) Guidelines [19, 20]. The protocol was registered in the PROSPERO database. Screening of articles, data extraction and quality assessment were independently undergoing by two reviewers. Any difference of opinions was discussed or adjudicated by a third reviewer.

### Data sources and search strategy

We systematically searched the studies compared RDG with LDG for GC published before December 31, 2021 in PubMed, Web of Science, Cochrane Library, and Embase database. The literature search formula in PubMed were (“Robotic Surgical Procedures”[Mesh]) AND (“Laparoscopy”[Mesh]) AND (“Gastrectomy”[Mesh]) and (“Robotic Surgical Procedures”[Mesh]) AND (“Laparoscopy”[Mesh]) AND (“Stomach Neoplasms”[Mesh]). The combination of free-text terms (robotic gastrectomy, laparoscopic gastrectomy, gastric cancer) was used in Web of Science, Cochrane Library, and Embase database. To find additional related studies, the references of eligible studies were manually searched.

### Inclusion and exclusion criteria

The included studies should meet the following criteria: (1) Studies conducted on adult patients who had been diagnosed as GC and underwent distal radical gastrectomy; (2) Comparative studies related to RDG and LDG; (3) At least 1 item of original data on interested perioperative outcomes could be obtained; (4) PSM or RCT studies; (5) Studies published in English.

The exclusion criteria: (1) Studies were not conducted on GC patients; (2) The data of studies was unavailable. (3) Neither PSM nor RCT studies.

### Data extraction and quality assessment

The original data of all the included articles were individually evaluated and extracted by two reviewers using a standardized datasheet. The collected data includes: name of first author, publication year, study design, study period, sample volume, age, extent of lymph node dissection, reconstruction methods, operative time, blood loss, retrieved lymph nodes, time to first flatus, time to first liquid intake, time to start soft diet, postoperative hospital stays, overall complications, complications Grade I–II, complications Grade ≥ III, anastomotic leakage, bleeding, intra-abdominal bleeding, intraluminal bleeding, ileus, abdominal infection, delayed gastric emptying, wound complications, pneumonia, cardiac complications, and urinary infection.

The ROBINS-I tool was used to assess the quality of PSM studies [21] and the Cochrane risk-of-bias tool for RCT studies [22]. The certainty of evidence for all the outcomes were assessed using the Grading of Recommendations, Assessment, Development and Evaluation (GRADE) approach [23].

### Statistical analysis

Review Manager 5.4 was used for statistical analyses. Mean difference (MD) with 95% confidence interval (CI) were used for continuous data, and Odds ratio (OR) with 95% CI for dichotomous data. Heterogeneity was assessed using the Chi-squared (χ^2^) and I-squared (*I*^2^) tests. A fixed-effects model (FEM) was used when the heterogeneity is low (*p* > 0.1 and *I*^2^ < 50%), otherwise a random-effects model (REM) was used. Publication bias was assessed using funnel plots and Egger’s test. A p-value less than 0.05 is statistically significant.

## Results

### Characteristics of the included studies

**Fig. 1:**
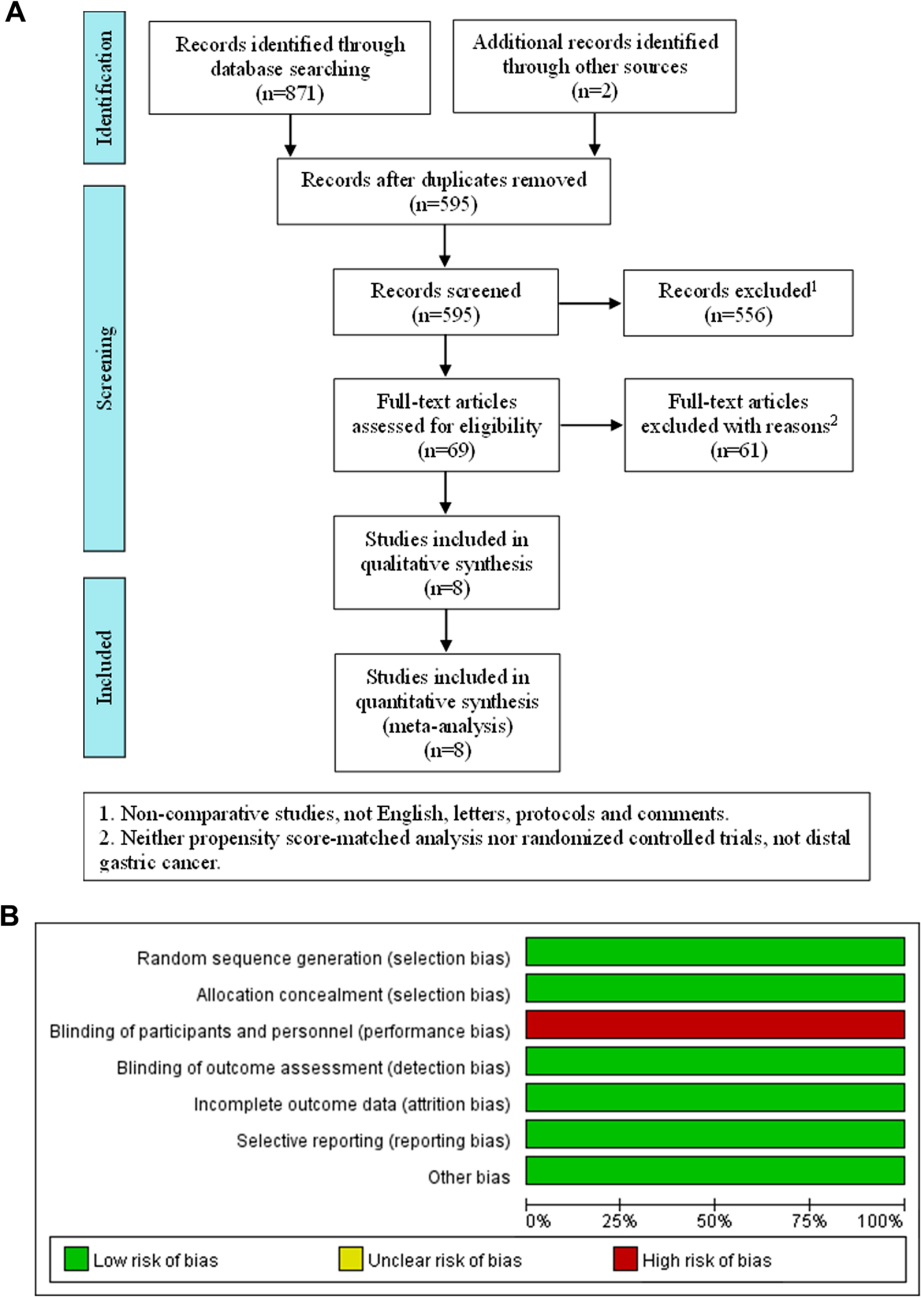
Flow diagram showing study selection process for meta-analysis (**A**); Risk of bias in RCT (**B**)

A total of 873 relevant English publications from various database were identified. 595 articles were filtered by titles and abstracts after duplicates removed. Then we obtained 69 articles for full-text assessment. According to the inclusion criteria, 7 PSM [24–30] studies and 1 RCT study [8] with a total of 2763 patients were finally included in this analysis. The flow diagram is showed in Fig. 1A, and Table 1 presents the individual characteristics of the selected studies. The risk of bias in PSM studies were assessed by ROBINS-I tool and present in Table 2. The risk of RCT was assessed by Cochrane risk-of-bias tool and present in Fig. 1B. The quality of evidence of every outcome was assessed by GRADE guideline and present in Table 3.

**Table 1:** Characteristics of included studies

Author year	Design	Study period	Volume		Age (mean ± SD)		LND	Reconstruction
			RDG	LDG	RDG	LDG		
Hong et al. 2016	PSM	2008–2015	232	232	53.7 ± 11.5	55.0 ± 13.0	D1, D1 + , D2	BI, BII, RY
Li et al. 2018	PSM	2013–2017	66	66	55.2 ± 11.6	54.1 ± 11.4	D2	BI, BII
Li et al. 2020	PSM	2010–2019	516	516	54.63 ± 11.85	55.10 ± 10.24	D1, D2	BI, BII
Roh et al. 2020	PSM	2015–2017	51	51	58.1 ± 10.8	58.0 ± 11.1	D1 +	BI, BII
Song et al. 2020	PSM	2016–2019	40	40	56.4 ± 12.8	58.1 ± 11.6	D2	BI, BII, RY
Ye et al. 2020	PSM	2014–2019	285	285	57.1 ± 8.3	57.0 ± 8.6	D2	BI, BII, RY
Isobe et al. 2021	PSM	2018–2020	50	50	69.2 ± 1.4	69.3 ± 1.4	D1, D1 + , D2	BI, BII, RY
Lu et al. 2021	RCT	2017–2020	141	142	59.4 ± 10.2	59.3 ± 11.3	D1 + , D2	BI, BII

*BI* Billroth I, *BII* Billroth II, *RY* Roux-en-Y

**Table 2:** Risk of bias in PSM studies (ROBINS-I)

Study	D1	D2	D3	D4	D5	D6	D7	Overall
Hong et al. [24]	Low	Moderate	Low	Low	Low	Low	Moderate	Moderate
Li et al. [25]	Low	Low	Low	Low	Low	Low	Moderate	Moderate
Li et al. [4]	Low	Moderate	Low	Low	Low	Low	Low	Moderate
Roh et al. [30]	Low	Low	Low	Low	Low	Low	Moderate	Moderate
Song et al. [27]	Low	Low	Low	Low	Low	Low	Moderate	Moderate
Ye et al. [28]	Low	Low	Low	Low	Low	Low	Low	Low
Isobe et al. [29]	Low	Moderate	Low	Low	Low	Low	Low	Moderate

Domains:

D1: Bias due to confounding

D2: Bias in selection of participants into the study

D3: Bias in classification of interventions

D4: Bias due to deviations from intended interventions

D5: Bias due to missing data

D6: Bias in measurement of outcomes

D7: Bias in selection of the reported result

**Table 3:** GRADE assessment for all the outcomes

Outcomes (No. of studies)	Certainty assessment						No. of patients	MD (95% CI) or OR (95% CI)
	Risk of bias	Inconsistency	Indirectness	Imprecision	Publication bias	Certainty		
Operative time (8)	Serious^a^	Not serious	Not serious	Serious^b^	None	Low	2763	33.22 [24.32, 42.11]
Blood loss (8)	Serious^a^	Not serious	Not serious	Serious^b^	None	Low	2763	− 28.56 [− 40.29, − 16.83]
Retrieved lymph nodes (7)	Serious^a^	Not serious	Not serious	Not serious	None	Moderate	2480	3.46 [2.94, 3.98]
Time to first flatus (6)	Serious^a^	Not serious	Not serious	Not serious	None	Moderate	2199	− 0.08 [− 0.13, − 0.02]
Time to first liquid intake (5)	Serious^a^	Not serious	Not serious	Not serious	None	Moderate	2481	− 0.13 [− 0.22, − 0.05]
Time to start soft diet (4)	Serious^a^	Not serious	Not serious	Not serious	None	Moderate	1246	− 0.04 [− 0.31, 0.23]
Hospital stays (8)	Serious^a^	Not serious	Not serious	Not serious	None	Moderate	2763	− 0.21 [− 0.44, 0.01]
Overall complications (8)	Serious^a^	Not serious	Not serious	Not serious	None	Moderate	2763	0.84 [0.68, 1.04]
Complications I–II (7)	Serious^a^	Not serious	Not serious	Not serious	None	Moderate	2631	0.91 [0.71, 1.18]
Complications ≥ III (7)	Serious^a^	Not serious	Not serious	Not serious	None	Moderate	2631	0.70 [0.48, 1.03]
Anastomotic leakage (5)	Serious^a^	Not serious	Not serious	Not serious	None	Moderate	2449	0.73 [0.29, 1.81]
Bleeding (5)	Serious^a^	Not serious	Not serious	Not serious	None	Moderate	2429	0.89 [0.46, 1.74]
Intra-abdominal bleeding (3)	Serious^a^	Not serious	Not serious	Not serious	None	Moderate	1885	0.54 [0.20, 1.48]
Intraluminal bleeding (3)	Serious^a^	Not serious	Not serious	Serious^b^	None	Low	1682	0.84 [0.27, 2.64]
Ileus (5)	Serious^a^	Not serious	Not serious	Not serious	None	Moderate	2429	0.85 [0.39, 1.88]
Abdominal infection (4)	Serious^a^	Not serious	Not serious	Not serious	None	Moderate	1985	0.91 [0.38, 2.15]
Delayed gastric emptying (4)	Serious^a^	Not serious	Not serious	Not serious	None	Moderate	1033	0.55 [0.18, 1.66]
Wound complications (5)	Serious^a^	Not serious	Not serious	Serious^b^	None	Low	2429	1.53 [0.77, 3.05]
Pneumonia (5)	Serious^a^	Not serious	Not serious	Not serious	None	Moderate	2065	0.66 [0.41, 1.05]
Cardiac complications (4)	Serious^a^	Not serious	Not serious	Serious^b^	None	Low	1965	1.81 [0.60, 5.43]
Urinary infection (4)	Serious^a^	Not serious	Not serious	Not serious	None	Moderate	1965	0.85 [0.27, 2.65]

^a^There may be implementation bias and measurement bias

^b^Wide range of 95% confidence intervals were identified

### Surgical outcomes: operative time, blood loss and retrieved lymph nodes

**Fig. 2:**
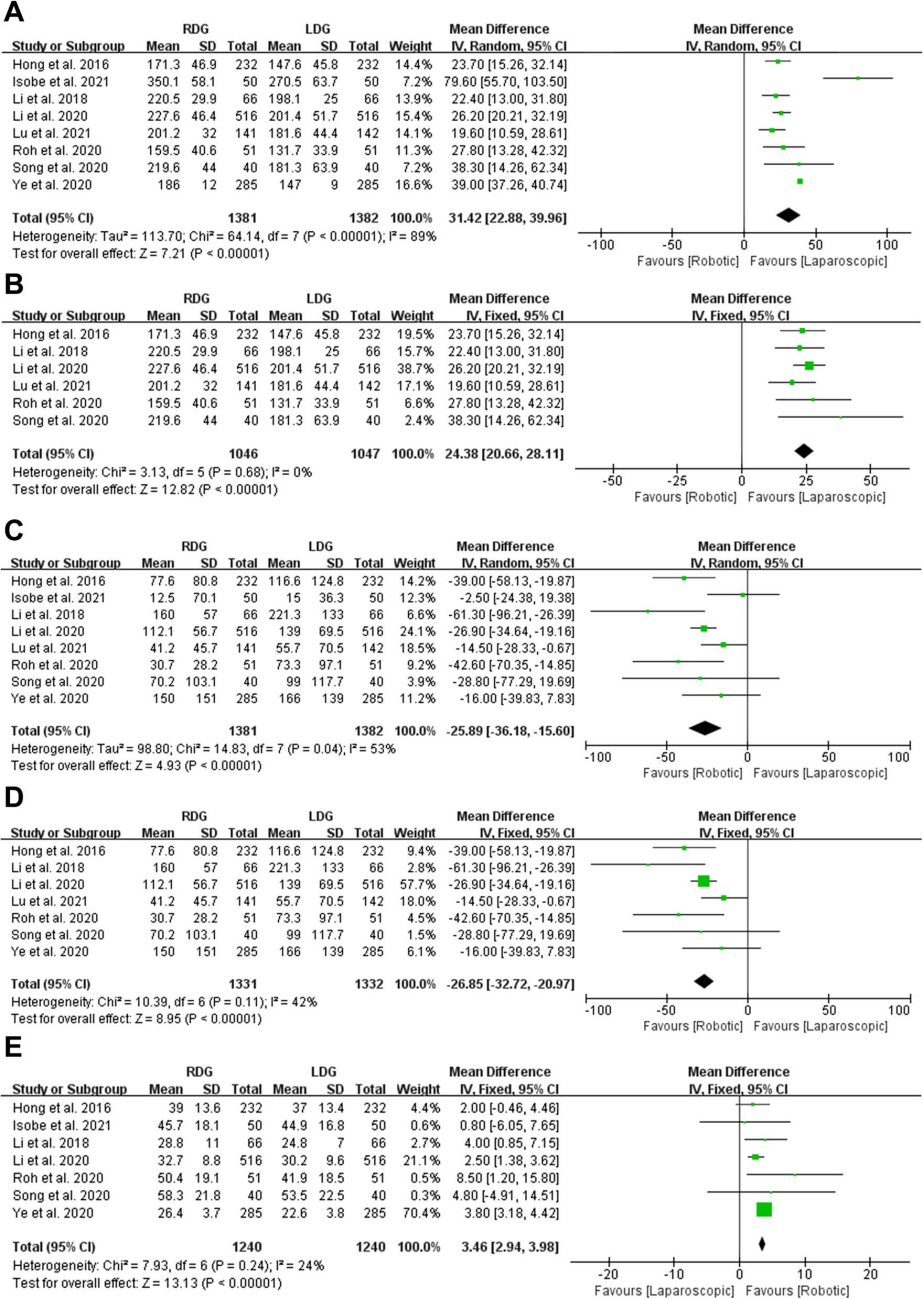
Forest plots of surgical outcomes: **A** operative time; **B** operative time without Isobe et al. [29] and Ye et al. [28]; **C** blood loss; **D** blood loss without Isobe et al. [29]; **E** retrieved lymph nodes

Eight studies evaluated the operative time with a total of 1381 patients in RDG group and 1382 patients in LDG groups. Due to the high heterogeneity on operative time in the eight studies (*p* < 0.00001, *I*^2^ = 89%), a REM was used. The present meta-analysis showed that operative time was longer in RDG group (MD = 31.42, 95% CI [22.88, 39.96], *p* < 0.00001) (Fig. 2A). Subsequently, we removed and re-entered each of these 8 studies in Review Manager 5.4 software, and found that the major reason for high heterogeneity lies with the studies of Isobe et al. [29] and Ye et al. [28]. Because the operative time in the studies of Isobe et al. [29] was obviously longer than other studies (350.1 ± 58.1 in RDG group, 270.5 ± 63.7 in LDG group, MD = 79.6), and a very narrow 95% CI was observed in the studies of Ye et al. [28] (95% CI [37.26, 40.74]). However, the statistically results in these two studies were agree with other six studies and our meta-analysis. The same result was obtained after a meta-analysis for the six studies (MD = 24.38, 95% CI [20.66, 28.11], p < 0.00001), heterogeneity was low (*p* = 0.68, *I*^2^ = 0%) and analyzed in FEM (Fig. 2B).

Eight studies with a total of 2763 patients reported blood loss. Due to the moderate heterogeneity (*p* = 0.04, *I*^2^ = 53%), a REM was used. The present meta-analysis showed that blood loss was less in RDG group (MD = − 25.89, 95% CI [− 36.18, − 15.6], *p* < 0.00001) (Fig. 2C). The moderate heterogeneity was owing to the less blood loss in the study of Isobe et al. [29] (12.5 ± 70.1 in RDG group, 15 ± 36.3 in LDG group, MD = − 2.5) than other seven studies. Furthermore, there is no significant difference in blood loss between RGD and LDG in the study of Isobe et al. [29] (*p* = 0.234), which was not consistent with other seven studies. Subsequently, a meta-analysis without the study of Isobe et al. [29] was performed, we observed that the blood loss was still less in RDG group (MD = − 26.85, 95% CI [− 32.72, − 20.97], p < 0.00001). Heterogeneity was low (*p* = 0.11, *I*^2^ = 42%) and analyzed in FEM (Fig. 2D).

Seven studies with a total of 2480 patients reported number of retrieved lymph nodes. Our meta-analysis suggested that the RDG group retrieved more lymph nodes than LDG group (MD = 3.46, 95% CI [2.94, 3.98], *p* < 0.00001). Heterogeneity was low (*p* = 0.24, *I*^2^ = 24%) and analyzed in FEM (Fig. 2E).

### Postoperative recovery: time to first flatus, time to first liquid intake, time to start soft diet and postoperative hospital stays

**Fig. 3:**
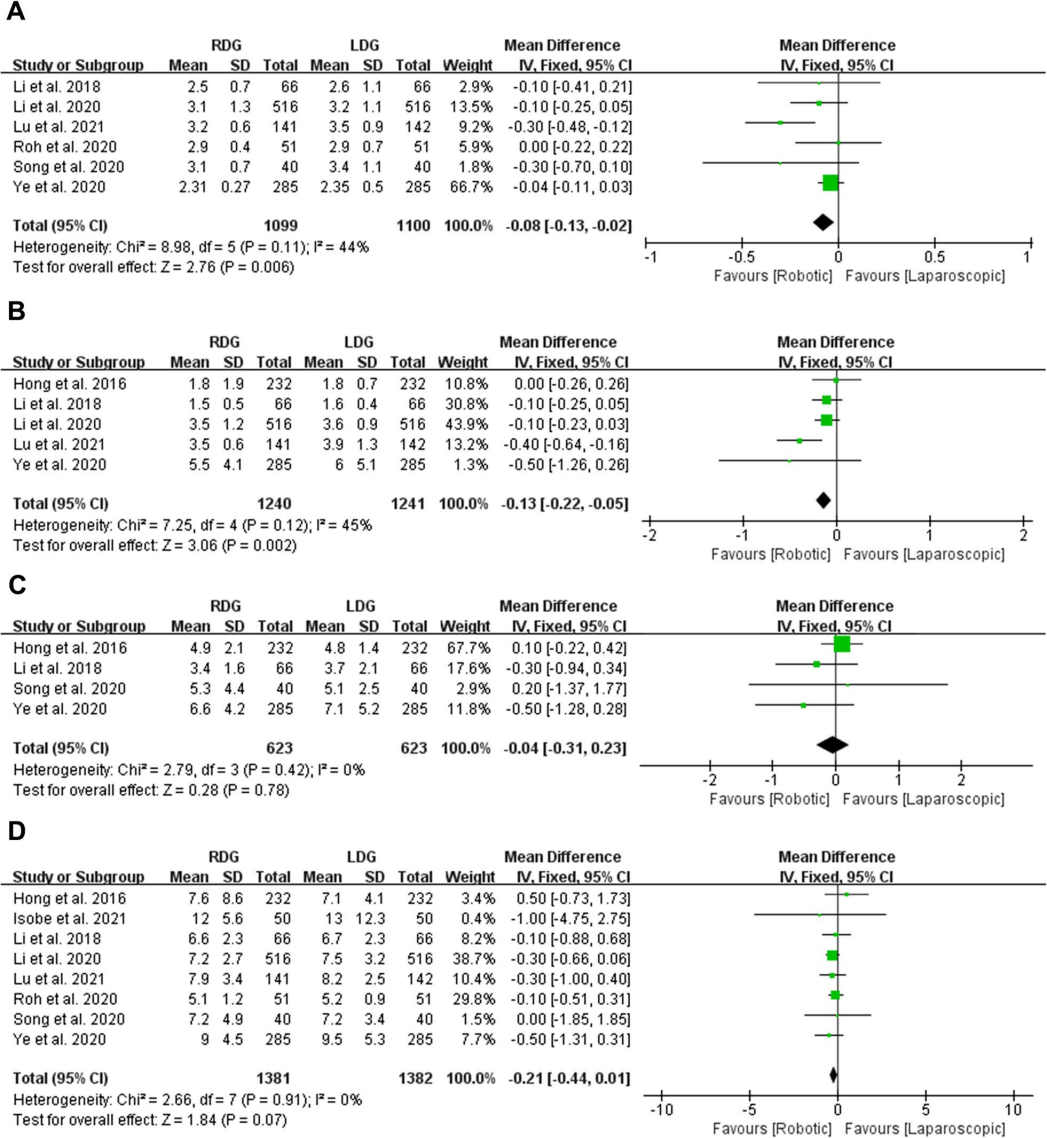
Forest plots of postoperative recovery: **A** time to first flatus; **B** time to first liquid intake; **C** time to start soft diet; **D** postoperative hospital stays

Six studies with a total of 2199 patients reported time to first flatus. Our meta-analysis using a FEM (Heterogeneity: *p* = 0.11, *I*^2^ = 44%) revealed that time to first flatus was less in RDG group than in LDG group (MD = − 0.08, 95% CI [− 0.13, − 0.02], *p* = 0.006) (Fig. 3A). Five studies with a total of 2481 patients reported time to first liquid intake. The meta-analysis using a FEM (*p* = 0.12, *I*^2^ = 45%) suggested less time to first liquid intake in RDG group (MD = − 0.13, 95% CI [− 0.22, − 0.05], *p* = 0.002) (Fig. 3B). Four studies with a total of 1246 patients reported time to start soft diet. No significant difference was observed between RDG and LDG groups (MD = − 0.04, 95% CI [− 0.31, 0.23], *p* = 0.78) after analyzed by FEM (*p* = 0.42, *I*^2^ = 0%) (Fig. 3C). All the studies with a total of 2763 patients reported length of postoperative hospital stays. No significant difference was observed between RDG and LDG groups (MD = − 0.21, 95% CI [− 0.44, 0.01], *p* = 0.07) after analyzed by FEM (Heterogeneity: *p* = 0.91, *I*^2^ = 0%) (Fig. 3D).

### Complications: overall complications, complications Grade I–II and complications Grade ≥ III

**Fig. 4:**
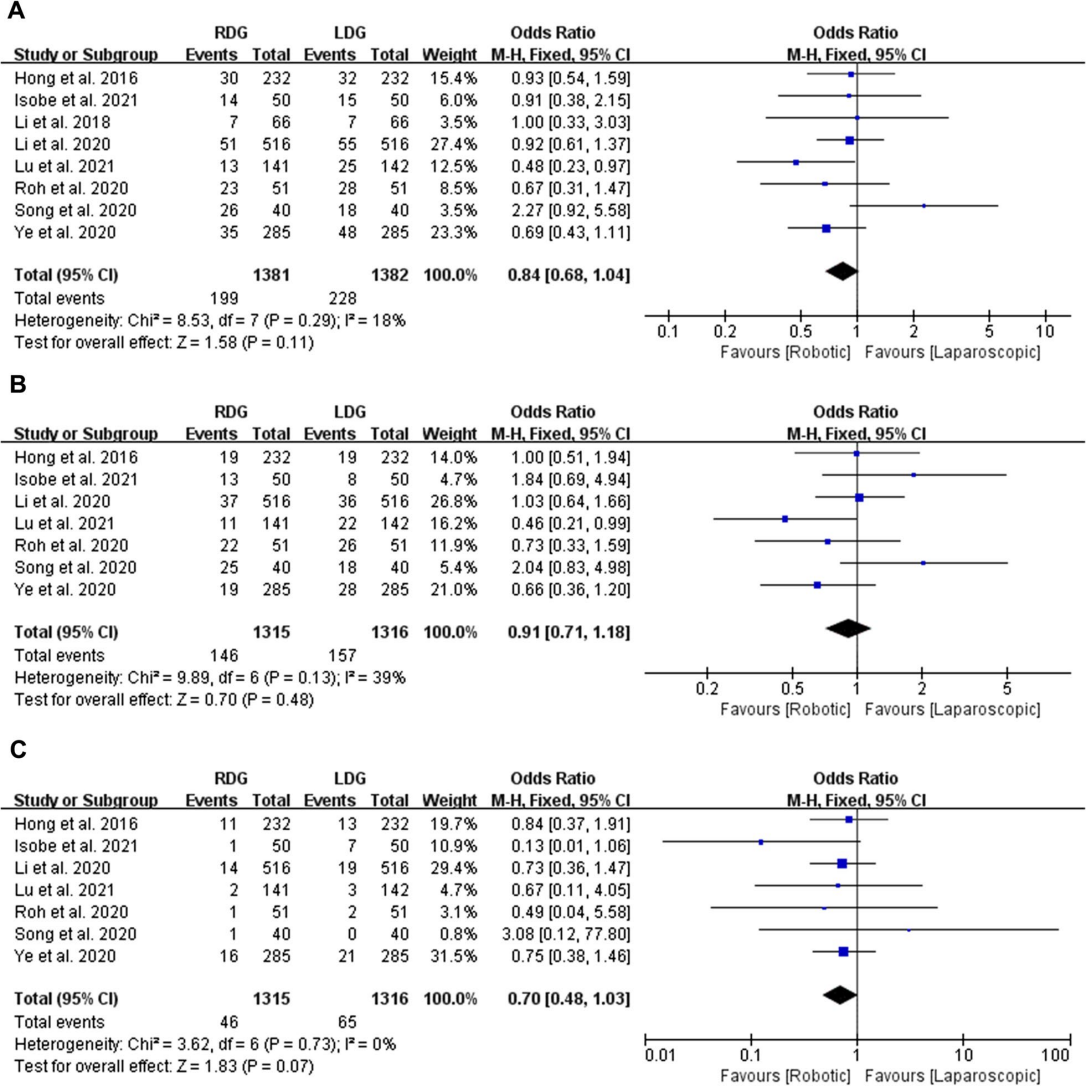
Forest plots of complications: **A** overall complications; **B** complications Grade I–II; **C** complications Grade ≥ III

All the studies with a total of 2763 patients reported overall complications. No significant difference was observed between the RDG and LDG groups (OR = 0.84, 95% CI [0.68, 1.04], *p* = 0.11) after analyzed by FEM (Heterogeneity: *p* = 0.29, *I*^2^ = 18%) (Fig. 4A). Seven studies with a total of 2631 patients stratified postoperative complications into five grades according to the Clavien–Dindo classification (CDC) [31–33]. Complications Grade I–II were considered as minor, Grade ≥ III were considered as severe complications. The present meta-analysis revealed no statistically significant in complications Grade I–II (OR = 0.91, 95% CI [0.71, 1.18], *p* = 0.48, heterogeneity: *p* = 0.13, *I*^2^ = 39%) and complications Grade ≥ III (OR = 0.70, 95% CI [0.48, 1.03], *p* = 0.07, heterogeneity: *p* = 0.73, *I*^2^ = 0%) between RDG and LDG groups (Fig. 4B, C).

### Surgical complications: anastomotic leakage, bleeding, intra-abdominal bleeding, intraluminal bleeding, ileus, abdominal infection, delayed gastric emptying and wound complications

#### Anastomotic leakage

**Fig. 5:**
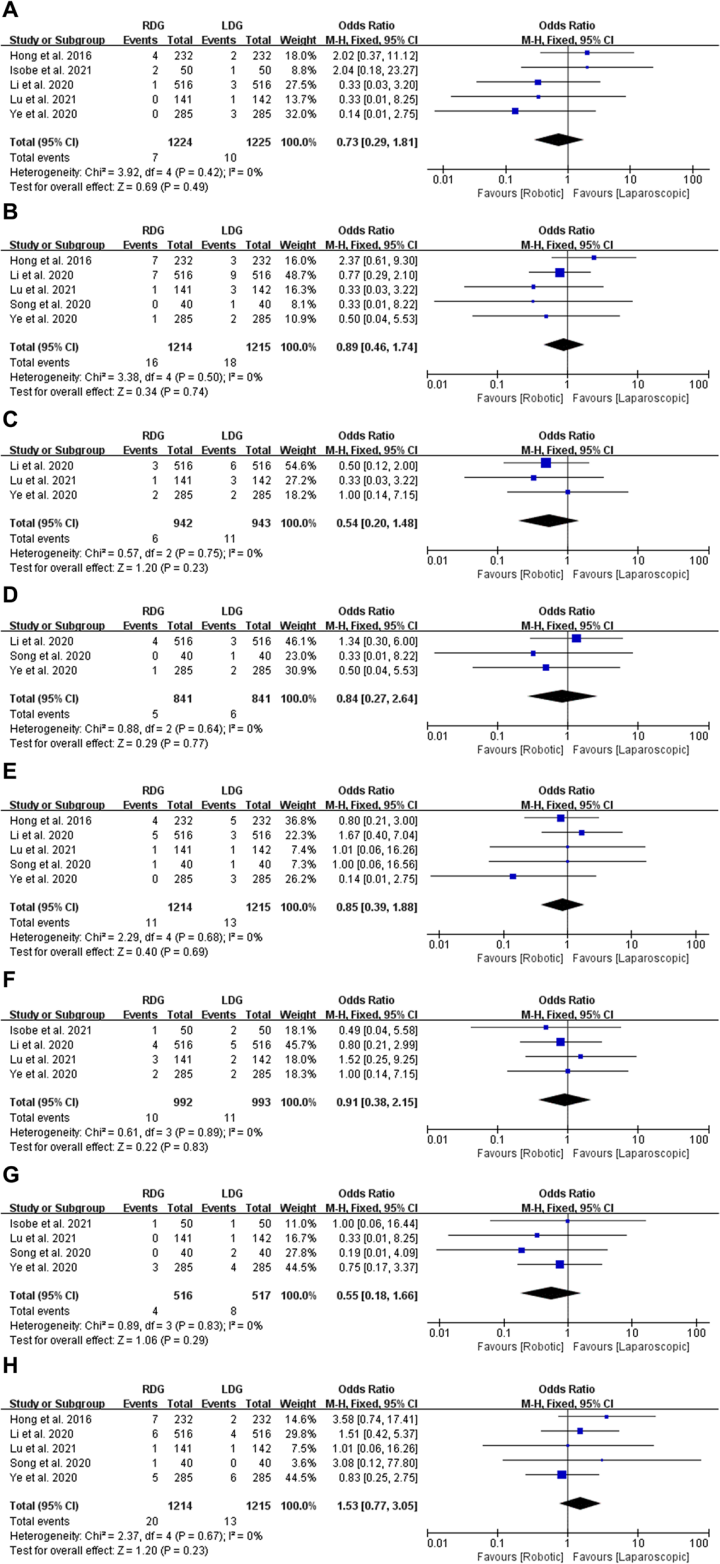
Forest plots of surgical complications: **A** anastomotic leakage; **B** bleeding; **C** intra-abdominal bleeding; **D** intraluminal bleeding; **E** ileus; **F** abdominal infection; **G** delayed gastric emptying; **H** wound complications

Five studies with a total of 2449 patients reported anastomotic leakage. The meta-analysis indicated that there is no statistically significant between RDG and LDG groups (OR = 0.73, 95% CI [0.29, 1.81], p = 0.49) with low heterogeneity (*p* = 0.42, *I*^2^ = 0%), and these were analyzed by FEM (Fig. 5A).

#### Bleeding, intra-abdominal bleeding and intraluminal bleeding

Of the eight studies, five, three and three studies provided data about bleeding, intra-abdominal bleeding and intraluminal bleeding, respectively. The present meta-analysis indicated that there are no statistically significant on these three items between RDG and LDG groups (OR = 0.89, 95% CI [0.46, 1.74], *p* = 0.74; OR = 0.54, 95% CI [0.20, 1.48], *p* = 0.23; OR = 0.84, 95% CI [0.27, 2.64], *p* = 0.77). All of them were analyzed by FEM, because of low heterogeneity (*p* = 0.50, *I*^2^ = 0%; *p* = 0.75, *I*^2^ = 0%; *p* = 0.64, *I*^2^ = 0%) (Fig. 5B–D).

#### Ileus

Five studies with a total of 2429 patients reported ileus. No significant difference was observed between RDG and LDG groups (OR = 0.85, 95% CI [0.39, 1.88], *p* = 0.69) after meta-analyzed by FEM (Heterogeneity: *p* = 0.68, *I*^2^ = 0%) (Fig. 5E).

#### Abdominal infection

Four studies with a total of 1985 patients reported abdominal infection. No significant difference was observed between RDG and LDG groups (OR = 0.91, 95% CI [0.38, 2.15], *p* = 0.83) after meta-analyzed by FEM (Heterogeneity: *p* = 0.89, *I*^2^ = 0%) (Fig. 5F).

#### Delayed gastric emptying

Four studies with a total of 1033 patients reported delayed gastric emptying. The meta-analysis showed no difference between RDG and LDG groups (OR = 0.55, 95% CI [0.18, 1.66], *p* = 0.29) with low heterogeneity (*p* = 0.83, *I*^2^ = 0%), and these were analyzed by FEM (Fig. 5G).

#### Wound complications

Five studies with a total of 2429 patients reported wound complications. The meta-analysis showed no difference between RDG and LDG groups (OR = 1.53, 95% CI [0.77, 3.05], *p* = 0.23) with low heterogeneity (*p* = 0.67, *I*^2^ = 0%), and these were analyzed by FEM (Fig. 5H).

### Systematic complications: pneumonia, cardiac complications and urinary infection

#### Pneumonia

**Fig. 6:**
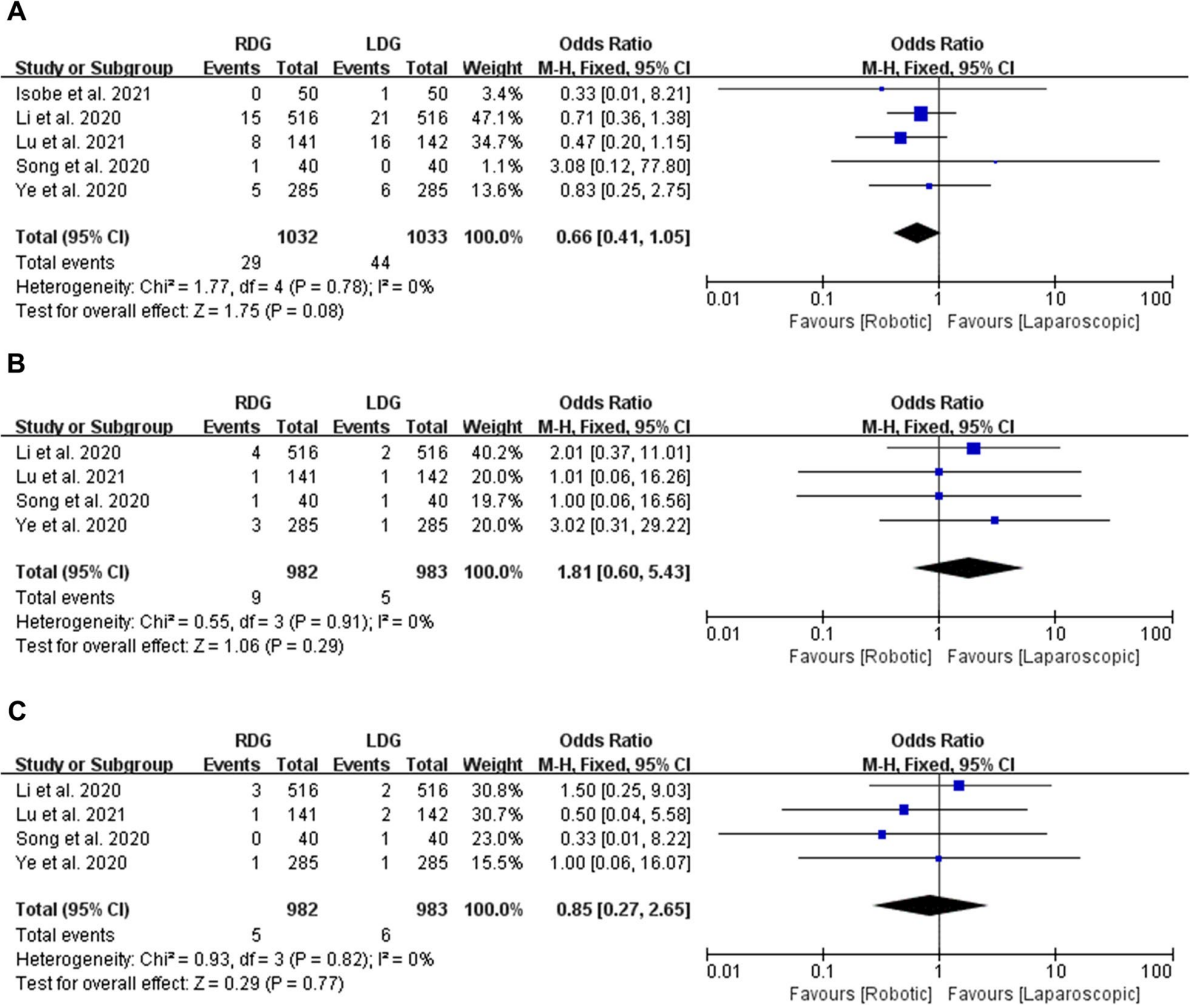
Forest plots of systematic complications: **A** pneumonia; **B** cardiac complications; **C** urinary infection

Five studies with a total of 2065 patients reported the incidence rate of pneumonia. The meta-analysis suggested that RDG had a similar incidence rate of pneumonia to that of the LDG group (OR = 0.66, 95% CI [0.41, 1.05], *p* = 0.08) after analyzed by FEM (Heterogeneity: *p* = 0.78, *I*^2^ = 0%) (Fig. 6A).

#### Cardiac complications

Four studies with a total of 1965 patients reported cardiac complications. No significant difference was observed between RDG and LDG groups (OR = 1.81, 95% CI [0.60, 5.43], *p* = 0.29) after meta-analyzed by FEM (Heterogeneity: *p* = 0.91, *I*^2^ = 0%) (Fig. 6B).

#### Urinary infection

Four studies with a total of 1965 patients provided data about urinary infection. The present meta-analysis indicated that there was no statistically significant between RDG and LDG groups (OR = 0.85, 95% CI [0.27, 2.65], *p* = 0.77). These were analyzed by FEM, because of low heterogeneity (*p* = 0.82, *I*^2^ = 0%) (Fig. 6C).

### Publication bias

**Fig. 7:**
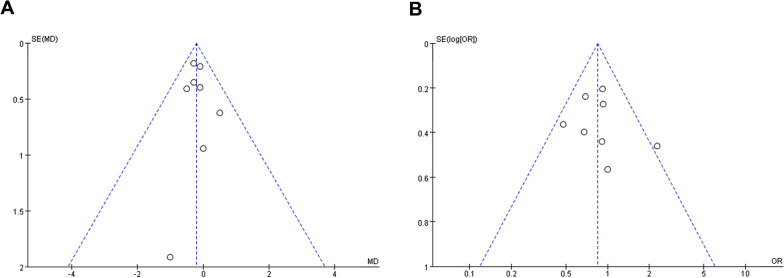
Funnel plot of postoperative hospital stays (**A**) and overall complications (**B**)

The potential role of publication bias was assessed by funnel plot. All studies lie inside the 95% CIs in the symmetrical funnel plot of postoperative hospital stays (Fig. 7A) and overall complications (Fig. 7B), indicating that there was no publication bias in these studies.

## Discussion

Minimally invasive is one of the important directions of surgery. Laparoscopic radical gastrectomy is currently the most widely used minimally invasive technique for GC. Several studies have proven the excellent surgical and oncological outcomes [34–36]. In recent years, a new form of surgery, robotic gastrectomy gradually developed. More and more studies have explored the safety and feasibility of robotic gastrectomy for GC [8, 11, 37]. However, their quality of evidence is jagged. Some studies compared the outcomes of RG and LG mixed different types of gastrectomy together [10, 24, 38]. But we all know that the extent of gastrectomy and lymph nodes dissection plays a critical role in the operative time, blood loss, number of retrieved lymph nodes, resection margin and incidence rate of various complications. Some studies reported the outcomes of small sample volume data and initial results in learning curve of robotic surgery [13–17]. The results of remaining big sample volume studies compared RDG with LDG were yet not entirely consistent. So that there are still controversies on the safety and efficacy of RDG in patients with GC. Therefore, a high-quality meta-analysis is necessary, and we performed it only included PSM and RCT studies focused on distal gastrectomy for the first time. In summary, the present study revealed that RDG has better surgical outcomes, faster postoperative recovery, and similar incidence rate of complications compared with LDG.

The present meta-analysis showed that the operative time was significantly longer in RDG group compared with LDG group. This is also a universal result, because the additional time, nearly half an hour, was required for docking and preparation [39]. The overall mean difference (MD = 31.42, 95% CI [22.88, 39.96]) revealed by the resent meta-analysis in operative time was coincided with the time for docking. Therefore, it would be impartial for RDG to calculate operative time after docking when compared with LDG, but most of studies didn’t do as this. Another important factor that affects the operative time is learning curve. Kim et al. [40] have reported that approximately 25 cases were needed to overcome operative time-learning curve sufficiently to gain proficiency for RG. Huang et al. [41] also reported that both operative time and docking time decreased and stabilized after 25 procedures for RG. The study performed by Li et al. [25] revealed that the operative time in RG was similar to that in LG after overcoming learning curve. Among the included studies in this meta-analysis, only one RCT indicated that all the RDG were performed by surgeons with experience of more than 50 robotic operations for GC before joining the trial [8]. One PSM study indicated that RDG were performed by one surgeon with experience of 14 robotic operations for GC [27]. As to the rest of studies, just a handful of initial results within learning curve were incorporated, and the proportion is very low. But to get more rigorous results, some large sample RCT studies that exclude the results within learning curve are still needed.

The meta-analysis revealed that RDG was associated with less blood loss and more retrieved lymph nodes. Manipulating in the correct anatomical gap and complete mesangial resection play a crucial role in reducing blood loss and ensuing the numbers of retrieved lymph nodes for radical gastrectomy [42]. The da Vinci vision system delivers highly magnified, 3D high-definition views of the surgical area, and it is more conducive to the identification of anatomical structures and gaps. The LDG included in the meta-analysis were performed using 2D laparoscopy with lowly magnified and 2D low-definition views. The visual difference between RDG and LDG should be the main reason for the statistically significant in blood loss and numbers of retrieved lymph nodes. Recently, 3D laparoscopy is widely used in surgical field. There is also study reported that 3D-LDG presented more retrieved lymph nodes and similar amount of blood loss [43]. Furthermore, the high degrees freedom EndoWrist and tremor filtering provided by da Vinci robotic surgery system benefit to the accuracy of operation, the bipolar Maryland forceps have a better hemostatic effect.

For postoperative recovery, our meta-analysis suggested that RDG had an earlier time to first flatus and liquid intake than LDG. However, there was no statistical differences in time to start soft diet and postoperative hospital stays between the two groups. Time to first flatus is an important indicator to reflect the recovery of gastrointestinal function after gastrectomy. Theoretically time to first liquid intake often consistent with time to first flatus. However, it would influence the accuracy of the two outcomes seriously along with the widespread development of enhanced recovery after surgery (ERAS) [44, 45], which was not indicated in the included studies. Habitually some medical institutions like to give patients soft diet or discharge after the risk period of anastomotic leakage, so that these two indicators were sometimes subjected to subjective decisions. But we still believe that RDG can accelerate the recovery of gastrointestinal function in patients with GC. These maybe owing to the reduced para-injury, and less pain, stress, inflammation result from precise manipulation in RDG group. The only RCT included in the meta-analysis also indicated that RDG could improve the postoperative recovery course [8].

When assessing the quality and safety of operations, postoperative complication is an important consideration. Here we counted the overall complications, surgical complications, and systemic complications. However, we did not find any differences between the two groups. We think this maybe result from that surgery in LDG group were performed by surgeons with extensive experience in laparoscopic surgery. The RDG group, on the other hand, contained data within the learning curve. Or the sample volume was not big enough to highlight the advantage of RDG.

Although all the studies included in the meta-analysis are high-quality evidences, 7 PSM studies and 1 RCT study, there are still some limitations in this study. First, only one RCT study compared RDG and LDG was published up to now, and was included in this meta-analysis. Second, the results in RDG group were influenced more or less by outcomes within learning curve. Third, this meta-analysis assessed the short-outcomes and safety of RDG versus LDG, however, the long-term oncological outcomes cannot be ignored in robotic surgery.

## Conclusion

In conclusion, RDG showed less blood loss and more retrieved lymph nodes, revealed less time to first flatus and liquid intake after operation. The incidence rate of postoperative complications was comparable between RDG and LDG. But the operative time in RDG was longer than LDG group, which we think was mainly delayed by docking. A much better result might be obtained in RDG group, if performed by surgeons mastered in robotic surgery.

## Data Availability

The datasets used and analyzed during the current study available from the corresponding author on reasonable request.

## Abbreviations

GC: Gastric cancer
RG: Robotic gastrectomy
LG: Laparoscopic gastrectomy
RCT: Randomized controlled trial
RDG: Robotic distal gastrectomy
LDG: Laparoscopic distal gastrectomy
PSM: Propensity score-matched
ERAS: Enhanced recovery after surgery

## References

[1] Wong MCS, Huang J, Chan PSF, Choi P, Lao XQ, Chan SM (2021). Global incidence and mortality of gastric cancer, 1980–2018. JAMA Netw Open.

[2] Japanese Gastric Cancer A (2021). Japanese gastric cancer treatment guidelines 2018 (5th edition). Gastr Cancer.

[3] Ajani JA, D'Amico TA, Bentrem DJ, Chao J, Cooke D, Corvera C (2022). Gastric cancer, Version 2. 2022, NCCN clinical practice guidelines in oncology. J Natl Compr Cancer Netw.

[4] Li J, Xi H, Guo X, Gao Y, Xie T, Qiao Z (2019). Surgical outcomes and learning curve analysis of robotic gastrectomy for gastric cancer: multidimensional analysis compared with threedimensional highdefinition laparoscopic gastrectomy. Int J Oncol.

[5] Furukawa T, Wakabayashi G, Ozawa S, Watanabe M, Ohgami M, Kitagawa Y (2000). Surgery using master–slave manipulators and telementoring. Nihon Geka Gakkai Zasshi.

[6] Mehrabi A, Yetimoglu CL, Nickkholgh A, Kashfi A, Kienle P, Konstantinides L (2006). Development and evaluation of a training module for the clinical introduction of the da Vinci robotic system in visceral and vascular surgery. Surg Endosc.

[7] Hashizume M, Shimada M, Tomikawa M, Ikeda Y, Takahashi I, Abe R (2002). Early experiences of endoscopic procedures in general surgery assisted by a computer-enhanced surgical system. Surg Endosc.

[8] Lu J, Zheng CH, Xu BB, Xie JW, Wang JB, Lin JX (2021). Assessment of robotic versus laparoscopic distal gastrectomy for gastric cancer: a randomized controlled trial. Ann Surg.

[9] Hosoda K, Mieno H, Ema A, Ushiku H, Washio M, Song I (2019). Safety and feasibility of robotic distal gastrectomy for stage IA gastric cancer: a phase II trial. J Surg Res.

[10] Kim HI, Han SU, Yang HK, Kim YW, Lee HJ, Ryu KW (2016). Multicenter prospective comparative study of robotic versus laparoscopic gastrectomy for gastric adenocarcinoma. Ann Surg.

[11] Ojima T, Nakamura M, Hayata K, Kitadani J, Katsuda M, Takeuchi A (2021). Short-term outcomes of robotic gastrectomy vs laparoscopic gastrectomy for patients with gastric cancer: a randomized clinical trial. JAMA Surg.

[12] Gong S, Li X, Tian H, Song S, Lu T, Jing W (2022). Clinical efficacy and safety of robotic distal gastrectomy for gastric cancer: a systematic review and meta-analysis. Surg Endosc.

[13] Pugliese R, Maggioni D, Sansonna F, Ferrari GC, Forgione A, Costanzi A (2009). Outcomes and survival after laparoscopic gastrectomy for adenocarcinoma. Analysis on 65 patients operated on by conventional or robot-assisted minimal access procedures. Eur J Surg Oncol.

[14] Cianchi F, Indennitate G, Trallori G, Ortolani M, Paoli B, Macri G (2016). Robotic vs laparoscopic distal gastrectomy with D2 lymphadenectomy for gastric cancer: a retrospective comparative mono-institutional study. BMC Surg.

[15] Noshiro H, Ikeda O, Urata M (2014). Robotically-enhanced surgical anatomy enables surgeons to perform distal gastrectomy for gastric cancer using electric cautery devices alone. Surg Endosc.

[16] Matsunaga T, Miyauchi W, Kono Y, Shishido Y, Miyatani K, Hanaki T (2020). The advantages of robotic gastrectomy over laparoscopic surgery for gastric cancer. Yonago Acta Med.

[17] Eom BW, Yoon HM, Ryu KW, Lee JH, Cho SJ, Lee JY (2012). Comparison of surgical performance and short-term clinical outcomes between laparoscopic and robotic surgery in distal gastric cancer. Eur J Surg Oncol.

[18] Austin PC, Stuart EA (2015). Moving towards best practice when using inverse probability of treatment weighting (IPTW) using the propensity score to estimate causal treatment effects in observational studies. Stat Med.

[19] Moher D, Shamseer L, Clarke M, Ghersi D, Liberati A, Petticrew M (2015). Preferred reporting items for systematic review and meta-analysis protocols (PRISMA-P) 2015 statement. Syst Rev.

[20] Page MJ, McKenzie JE, Bossuyt PM, Boutron I, Hoffmann TC, Mulrow CD (2021). The PRISMA 2020 statement: an updated guideline for reporting systematic reviews. Int J Surg.

[21] Sterne JA, Hernan MA, Reeves BC, Savovic J, Berkman ND, Viswanathan M (2016). ROBINS-I: a tool for assessing risk of bias in non-randomised studies of interventions. BMJ.

[22] Higgins JP, Altman DG, Gotzsche PC, Juni P, Moher D, Oxman AD (2011). The Cochrane Collaboration's tool for assessing risk of bias in randomised trials. BMJ.

[23] Balshem H, Helfand M, Schunemann HJ, Oxman AD, Kunz R, Brozek J (2011). GRADE guidelines: 3. Rating the quality of evidence. J Clin Epidemiol.

[24] Hong SS, Son SY, Shin HJ, Cui LH, Hur H, Han SU (2016). Can robotic gastrectomy surpass laparoscopic gastrectomy by acquiring long-term experience? A propensity score analysis of a 7-year experience at a single institution. J Gastr Cancer.

[25] Li Z, Li J, Li B, Bai B, Liu Y, Lian B (2018). Robotic versus laparoscopic gastrectomy with D2 lymph node dissection for advanced gastric cancer: a propensity score-matched analysis. Cancer Manage Res.

[26] Zheng-Yan L, Yong-Liang Z, Feng Q, Yan S, Pei-Wu Y (2021). Morbidity and short-term surgical outcomes of robotic versus laparoscopic distal gastrectomy for gastric cancer: a large cohort study. Surg Endosc.

[27] Song JH, Son T, Lee S, Choi S, Cho M, Kim YM (2020). D2 lymph node dissections during reduced-port robotic distal subtotal gastrectomy and conventional laparoscopic surgery performed by a single surgeon in a high-volume center: a propensity score-matched analysis. J Gastr Cancer.

[28] Ye SP, Shi J, Liu DN, Jiang QG, Lei X, Tang B (2020). Robotic- versus laparoscopic-assisted distal gastrectomy with D2 lymphadenectomy for advanced gastric cancer based on propensity score matching: short-term outcomes at a high-capacity center. Sci Rep.

[29] Isobe T, Murakami N, Minami T, Tanaka Y, Kaku H, Umetani Y (2021). Robotic versus laparoscopic distal gastrectomy in patients with gastric cancer: a propensity score-matched analysis. BMC Surg.

[30] Roh CK, Choi S, Seo WJ, Cho M, Choi YY, Son T (2020). Comparison of surgical outcomes between integrated robotic and conventional laparoscopic surgery for distal gastrectomy: a propensity score matching analysis. Sci Rep.

[31] Dindo D, Demartines N, Clavien PA (2004). Classification of surgical complications: a new proposal with evaluation in a cohort of 6336 patients and results of a survey. Ann Surg.

[32] Katayama H, Kurokawa Y, Nakamura K, Ito H, Kanemitsu Y, Masuda N (2016). Extended Clavien–Dindo classification of surgical complications: Japan Clinical Oncology Group postoperative complications criteria. Surg Today.

[33] Clavien PA, Barkun J, de Oliveira ML, Vauthey JN, Dindo D, Schulick RD (2009). The Clavien-Dindo classification of surgical complications: five-year experience. Ann Surg.

[34] Liu F, Huang C, Xu Z, Su X, Zhao G, Ye J (2020). Morbidity and mortality of laparoscopic vs open total gastrectomy for clinical stage I gastric cancer: the CLASS02 multicenter randomized clinical trial. JAMA Oncol.

[35] Hyung WJ, Yang HK, Park YK, Lee HJ, An JY, Kim W (2020). Long-term outcomes of laparoscopic distal gastrectomy for locally advanced gastric cancer: the KLASS-02-RCT randomized clinical trial. J Clin Oncol.

[36] Hu Y, Huang C, Sun Y, Su X, Cao H, Hu J (2016). Morbidity and mortality of laparoscopic versus open D2 distal gastrectomy for advanced gastric cancer: a randomized controlled trial. J Clin Oncol.

[37] Kim YM, Hyung WJ (2021). Current status of robotic gastrectomy for gastric cancer: comparison with laparoscopic gastrectomy. Updates Surg.

[38] Han DS, Suh YS, Ahn HS, Kong SH, Lee HJ, Kim WH (2015). Comparison of surgical outcomes of robot-assisted and laparoscopy-assisted pylorus-preserving gastrectomy for gastric cancer: a propensity score matching analysis. Ann Surg Oncol.

[39] Kandil EH, Noureldine SI, Yao L, Slakey DP (2012). Robotic transaxillary thyroidectomy: an examination of the first one hundred cases. J Am Coll Surg.

[40] Kim MS, Kim WJ, Hyung WJ, Kim HI, Han SU, Kim YW (2021). Comprehensive learning curve of robotic surgery: discovery from a multicenter prospective trial of robotic gastrectomy. Ann Surg.

[41] Huang KH, Lan YT, Fang WL, Chen JH, Lo SS, Li AF (2014). Comparison of the operative outcomes and learning curves between laparoscopic and robotic gastrectomy for gastric cancer. PLoS ONE.

[42] Xu Y, Li Z, Pan G, Wu H, Li J, Lin W (2021). Anatomical findings and short-term efficacy of fascial anatomy-guided infrapyloric lymphadenectomy in laparoscopic radical gastrectomy for gastric cancer. Surg Laparosc Endosc Percutan Tech.

[43] Cui H, Liu GX, Deng H, Cao B, Zhang W, Liang WQ (2020). Comparison of short-term efficacy between robotic and 3D laparoscopic-assisted D2 radical distal gastrectomy for gastric cancer. Zhonghua Wei Chang Wai Ke Za Zhi.

[44] Mortensen K, Nilsson M, Slim K, Schafer M, Mariette C, Braga M (2014). Consensus guidelines for enhanced recovery after gastrectomy: Enhanced Recovery After Surgery (ERAS(R)) Society recommendations. Br J Surg.

[45] Pedziwiatr M, Mavrikis J, Witowski J, Adamos A, Major P, Nowakowski M (2018). Current status of enhanced recovery after surgery (ERAS) protocol in gastrointestinal surgery. Med Oncol.

